# Toward Ultrasensitive Electrochemical Detection of Ammonia Nitrogen in Drinking Water: PtCo Alloy Nanosheet on Self-Supported Carbon Cloth

**DOI:** 10.3390/s26103103

**Published:** 2026-05-14

**Authors:** Ziyi Zhuang, Liang Jia, Cong Zhao, Zhiyun An, Jiameng Chen, Chun Zhao, Hui Suo

**Affiliations:** 1 State Key Laboratory of Integrated Optoelectronics, College of Electronic Science and Engineering, Jilin University, Changchun 130012, China; zhuangzy24@mails.jlu.edu.cn (Z.Z.); liangjia25@mails.jlu.edu.cn (L.J.); 17390030123@163.com (Z.A.); chenjm9924@mails.jlu.edu.cn (J.C.); zchun@jlu.edu.cn (C.Z.); 2 Jilin Province Product Quality Supervision and Inspection Institute, No.2699, YiJu Road, Changchun 130103, China; zhaocong@jlzjy.org

**Keywords:** electrochemical sensor, PtCo alloy nanosheet, ammonia nitrogen, water quality monitoring

## Abstract

Ammonia nitrogen is a key indicator for evaluating drinking water quality, and its accurate determination is of great significance for environmental monitoring and public health protection. In this work, a self-supported electrochemical sensor based on PtCo alloy nanosheets was fabricated on carbon cloth via a one-step electrodeposition strategy. The nanosheet structure facilitates the exposure of abundant electroactive sites and promotes efficient electron transfer. Electrochemical measurements indicate that the PtCo/CC electrode exhibits higher electrocatalytic activity toward ammonia oxidation than monometallic Pt, which can be attributed to the modulation of the Pt electronic structure induced by Co incorporation. Under linear sweep voltammetry, the optimized electrode exhibits a high sensitivity of 32.94 μA μM^−1^ cm^−2^ in the concentration range of 0.7–10 μM and 11.43 μA μM^−1^ cm^−2^ in the range of 10–100 μM, with a low detection limit of 77.9 nM. In addition, the electrode maintains good selectivity in the presence of common interfering ions, along with satisfactory reproducibility and stability. The feasibility of practical application is further confirmed by real water sample analysis. Overall, this work provides an effective strategy for the design of Pt-based alloy electrodes for ammonia nitrogen detection, with potential applications in drinking water quality monitoring.

## 1. Introduction

Ammonia nitrogen is a key nitrogen-containing pollutant in water bodies and is currently widely regarded as one of the primary indicators for assessing the quality of aquatic environments and drinking water [1,2]. Ammonia nitrogen in water bodies primarily originates from pesticides, chemical fertilisers, domestic sewage discharge and industrial effluents; its excessive presence not only leads to eutrophication but may also have serious impacts on aquatic ecosystems [3,4]. More importantly, within drinking water distribution systems, high concentrations of ammonia nitrogen can lead to a reduction in the efficacy of disinfectants and the regrowth of microorganisms, causing water quality issues and posing a serious threat to drinking water safety [5]. Consequently, the World Health Organisation (WHO) drinking water standards list it as one of the chemical parameters of concern in drinking water treatment, aiming to highlight its impact on disinfection efficacy and water quality stability in distribution networks [6]. To safeguard drinking water safety, China’s “Standards for Drinking Water Quality” (GB 5749-2022) stipulates that the concentration of ammonia nitrogen in drinking water must not exceed 0.5 mg L^−1^ [7]. Consequently, the development of rapid, reliable and highly sensitive ammonia nitrogen detection techniques is of great significance for ensuring water quality safety.

Currently, methods for detecting ammonia nitrogen primarily include spectrophotometry [8], fluorescence analysis [9], colorimetric analysis [10] and electrochemical detection methods [11]. Among these, electrochemical detection technology has attracted widespread attention due to its advantages of high sensitivity, rapid response, simple operation and suitability for on-site monitoring. In particular, Pt-based electrocatalytic electrochemical sensors demonstrate excellent catalytic activity in the ammonia oxidation reaction, effectively promoting the electrochemical ammonia oxidation process and enabling sensitive detection of ammonia nitrogen [12]. The O-S theory proposed by Oswin and Salomon and the G-M theory proposed by Gerischer and Mauerer effectively explain the reaction pathways of Pt-catalysed ammonia oxidation [13]. Oswin and Salomon propose that NH_3_ in aqueous solution adsorbs onto the Pt surface to form *NH_3_. This compound then undergoes continuous dehydrogenation to form various active intermediates (*NH_2_, *NH and *N), ultimately yielding N_2_ [14]. In contrast, Gerischer and Mauerer suggest that *NH_3_ undergoes dehydrogenation to form *N_2_H_x+y_, which subsequently undergoes further dehydrogenation to generate N_2_ [15]. Based on the above mechanistic analysis, ammonia oxidation involves a multi-step ammonia dehydrogenation process with a high reaction energy barrier, which limits the overall reaction efficiency; simultaneously, the ammonia oxidation reaction is susceptible to adsorption poisoning by intermediates, leading to a decline in catalytic activity.

To address the above issues, forming alloys of Pt with transition metals is considered an effective strategy to reduce the energy barrier of the multi-step ammonia dehydrogenation reaction and mitigate Pt poisoning [16]. In recent years, researchers have conducted extensive explorations on Pt-based alloys for electrochemical ammonia nitrogen detection. In 2022, Wang et al. synthesized PtCu binary alloy nanoparticles in situ on carbon cloth; the introduction of Cu enabled the electrode to achieve a sensitivity of 9.5 μA μM^−1^ cm^−2^ for ammonia nitrogen detection, with a detection limit of 8.6 nM [17]. In 2025, Wang et al. synthesized PtZnCu nanoalloys in situ on carbon cloth; leveraging the synergistic effect of Zn and Cu to modulate the electronic structure of Pt, the sensitivity was enhanced to 23.9 μA μM^−1^ cm^−2^, with a detection limit as low as 8.37 nM [18]. In the same year, Han et al. fabricated PtNi alloy nanosheets on carbon cloth via electrodeposition; the introduction of Ni resulted in an electrode sensitivity of 10.2 μA μM^−1^ cm^−2^ and a detection limit of 1.154 μM [19]. These studies indicate that alloying Pt with transition metals can effectively tune its electronic structure, thereby significantly enhancing ammonia nitrogen detection performance. However, the modulatory effects of different transition metals on the electronic structure of Pt vary considerably. Therefore, further investigation is needed to explore the influence of different transition metals on the electronic structure regulation of Pt and their subsequent impact on ammonia nitrogen detection performance.

Among the many transition metals, cobalt (Co) has attracted considerable attention due to its unique electronic structure, abundant resources and relatively low cost. Research has shown that the introduction of Co can induce strong electronic interactions with Pt, modulating the centre of the Pt d-band and improving reaction kinetics, thereby enhancing overall catalytic activity. Consequently, PtCo alloys demonstrate outstanding performance in various electrochemical reactions: in 2012, Choi et al. enhanced the activity of the oxygen reduction reaction by modulating the composition of PtCo alloy nanocubes, thereby altering the electronic structure and lattice contraction of the Pt alloy nanocatalyst [20]. In 2018, Luo et al. constructed a Pt-skin structure with high-index crystal faces through near-surface engineering of PtCo nanowires, which not only enabled real-time monitoring of H_2_O_2_ release from living cells but also demonstrated excellent sensing performance for molecules such as NH_2_NH_2_, dopamine and paracetamol [21]. In 2023, Yan et al. loaded PtCo alloy nanoparticles onto nitrogen-doped porous carbon; the doping of Co caused the d-band centre of the PtCo alloy to shift downwards, thereby optimising the adsorption free energy of H* and OH* and enhancing hydrogen evolution performance [22]. Although significant progress has been made with PtCo alloys in oxygen reduction, hydrogen evolution, and the sensing of molecules such as H_2_O_2_ and NH_2_NH_2_, their application in the electrochemical detection of ammonia nitrogen in aqueous solutions remains rarely reported. Consequently, extending the use of PtCo alloys to the field of ammonia nitrogen sensing holds great potential for practical application.

On the other hand, the electrode substrate material also plays a crucial role in the performance of electrochemical sensors. Carbon cloth (CC) is widely used in self-supporting electrode systems due to its excellent conductivity, good flexibility and three-dimensional porous structure [23]. Its porous structure not only facilitates electrolyte diffusion but also provides abundant loading sites, thereby enhancing sensitivity. Furthermore, carbon cloth exhibits good chemical stability, including resistance to acid and alkali corrosion and durability under harsh conditions, making it suitable for electrochemical sensing applications across a wide range of pH conditions. Consequently, combining PtCo alloy with a carbon cloth substrate holds promise for the development of a high-performance electrochemical sensing platform.

Based on the aforementioned research background, this work employs a one-step electrodeposition technique to successfully develop a highly sensitive ammonia nitrogen sensing platform for drinking water quality assessment: a self-supporting carbon cloth electrode modified with PtCo alloy nanosheets (PtCo/CC). Through in situ growth, this electrode forms a three-dimensional conductive network structure rich in active sites, which provides an efficient reaction interface for the ammonia oxidation reaction. Owing to the synergistic catalytic effect between Pt and Co in the PtCo/CC electrode, combined with the excellent electrical conductivity of the carbon cloth, the electrode exhibits strong electrocatalytic activity in the ammonia oxidation reaction, thereby enabling highly sensitive and selective detection of ammonia nitrogen. This study provides an effective strategy for the rapid detection of ammonia nitrogen in drinking water and demonstrates promising application prospects in the field of water quality monitoring.

## 2. Materials and Methods

### 2.1. Chemical Reagent

All chemical reagents used in this experiment are of analytical grade and do not require further purification. Sodium bicarbonate (NaHCO_3_), cobalt chloride hexahydrate (CoCl_2_·6H_2_O), potassium chloride (KCl), sodium nitrite (NaNO_2_), and potassium ferricyanide (K_3_[Fe(CN)_6_]) were all purchased from Beijing Chemical Works Co., Ltd. (Beijing, China). Ammonium chloride (NH_4_Cl), chloroplatinic acid hexahydrate (H_2_PtCl_6_·6H_2_O), potassium hydroxide (KOH), dipotassium phosphate trihydrate (K_2_HPO_4_·3H_2_O), and potassium dihydrogen phosphate (KH_2_PO_4_) were all purchased from Sinopharm Chemical Reagent Co., Ltd. (Shanghai, China). Magnesium sulfate heptahydrate (MgSO_4_·7H_2_O) was purchased from Tianjin Damiao Chemical Reagent Factory (Tianjin, China). Anhydrous sodium carbonate (Na_2_CO_3_), toluene (C_6_H_5_CH_3_), and acetone (CH_3_COCH_3_) were all purchased from Xilong Chemical Co., Ltd. (Shantou, China).

### 2.2. Synthesis of PtCo/CC Electrodes

Cut the carbon cloth into 0.5 cm × 1.5 cm pieces, then clean them by ultrasonication sequentially in toluene, acetone, ethanol, and deionized water for 30 min each. After cleaning, place the carbon cloth in an oven and dry at 60 °C. To enhance the hydrophilicity of the carbon cloth, prepare a mixed acid solution with a volume ratio of concentrated nitric acid to concentrated sulfuric acid of 3:1. Place the dried carbon cloth in the mixed acid solution for 72 h, then rinse repeatedly with deionized water until the rinsing solution reaches a neutral pH. Finally, dry it in an oven. The carbon cloth treatment is thus complete.

In this study, an electrochemical method was employed to prepare a PtCo/CC electrode in a three-electrode system. The counter electrode consisted of a 1.5 cm × 1.5 cm platinum sheet, the reference electrode was a silver/silver chloride (Ag/AgCl) electrode, and the working electrode was the carbon cloth treated as described above (the area exposed to the precursor solution was 0.5 cm × 1 cm). The precursor solution was prepared using 25 mL of deionised water as the solvent, to which 0.0125 mmol of cobalt chloride hexahydrate and 0.0875 mmol of chloroplatinic acid hexahydrate were added as solutes, resulting in a total molar concentration of 4 mM. The aforementioned three-electrode system was placed in the precursor solution, and deposition was carried out using cyclic voltammetry with the following parameters: a potential window of −0.8 V to 0.6 V (vs. Ag/AgCl), a scan rate of 50 mV/s, and 55 cycles. Upon completion of the deposition, the electrode was rinsed thoroughly with deionised water and dried in an oven to yield the PtCo/CC electrode. By way of comparison, a Pt/CC electrode was prepared using the same cyclic voltammetry method. The precursor solution consisted of 0.1 mmol of chloroplatinic acid hexahydrate dissolved in 25 mL of deionised water, with a concentration of 4 mM. The three-electrode system was placed in this precursor solution, and the electrode was prepared using exactly the same electrochemical deposition parameters as for the PtCo/CC electrode. Upon completion of the deposition, the electrode was rinsed with deionised water and dried to yield the Pt/CC electrode.

### 2.3. Characterization and Electrochemical Testing

A scanning electron microscope (SEM, JEOL-JEM-6700F, JEOL, Ltd., Tokyo, Japan) was used to analyse the morphology and structure of the prepared electrodes. An X-Ray Diffractometer (XRD, D8ADVANCE, Bruker, Karlsruhe, Germany, Cu Kα source, λ = 1.54 Å) was employed to analyse the crystal structure of the prepared electrodes. An X-ray photoelectron spectrometer (XPS, ESCALAB-250, Thermo Fisher, Waltham, MA, USA) was used to qualitatively analyse the chemical composition and valence states of the prepared electrodes.

All electrochemical measurements were performed on an electrochemical workstation (CHI 760D, Shanghai Chenhua Instrument Co., Shanghai, China). For the electrochemical performance tests using the ferri/ferrocyanide probe, the electrolyte was 0.1 M KCl containing 5 mM [Fe(CN)_6_]^3−/4−^, the reference electrode was Ag/AgCl (saturated KCl as internal electrolyte), the counter electrode was a 1.5 cm × 1.5 cm Pt sheet, and the working electrode was the electrode under test. Cyclic voltammetry was employed, with the following parameters: potential window of −0.1 V to 0.6 V (vs. Ag/AgCl) and scan rate ranging from 20 to 200 mV/s. For ammonia nitrogen detection, the electrolyte was 1 M KOH, the reference electrode was Hg/HgO (1 M KOH as internal electrolyte), the counter electrode was a 1.5 cm × 1.5 cm Pt sheet, and the working electrode was the as-prepared PtCo/CC electrode. Ammonia nitrogen was analysed using cyclic voltammetry and linear sweep voltammetry. The cyclic voltammetry parameters were a potential window of −0.6 V to 0 V (vs. Hg/HgO) and a scan rate of 50 mV/s; the linear sweep voltammetry parameters were a potential window of −0.5 V to −0.1 V (vs. Hg/HgO) and a scan rate of 50 mV/s. It should be noted that, because the electrolytes used in the two test systems are different, different reference electrodes compatible with each electrolyte were adopted (Ag/AgCl for the KCl system and Hg/HgO for the KOH system). All potentials are reported directly with respect to the corresponding reference electrode, and no conversion between the two reference electrodes was performed.

## 3. Results and Discussion

### 3.1. Structural and Morphological Characterisation

**Figure 1:**
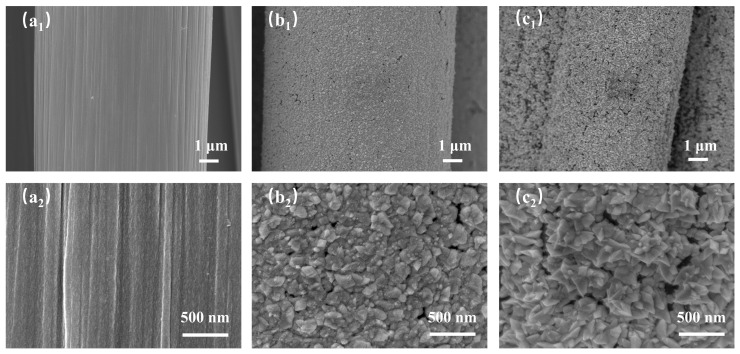
(**a1**,**a2**) SEM images of CC at low and high magnifications; (**b1**,**b2**) SEM images of Pt/CC at low and high magnifications; (**c1**,**c2**) SEM images of PtCo/CC at low and high magnifications.

SEM was employed to systematically characterise the surface morphology of the prepared PtCo/CC electrode, Pt/CC electrode, and bare carbon cloth. As shown in Figure 1(a1,a2), the surface of the raw carbon cloth appears relatively smooth at low magnification, whilst at high magnification it exhibits a distinct fibre groove structure, indicating that it primarily serves as a conductive substrate with limited active sites. Following Pt loading, as shown in Figure 1(b1,b2), the surface of the Pt/CC electrode exhibits a relatively uniform coating at low magnification; however, high-magnification observation reveals a nanoparticle morphology. Although the particles are distributed relatively uniformly, a certain degree of agglomeration is present, resulting in a relatively dense structure and a reduced effective specific surface area, which may consequently limit its electrocatalytic performance. As shown in Figure 1(c1,c2), at low magnification, PtCo is seen to cover the carbon fibre surface uniformly, with no large exposed areas; high-magnification observation further reveals the growth of a large number of ultra-thin nanosheets on the surface, with individual nanosheets having a lateral dimension of approximately 200–500 nm. These nanosheets are interwoven and stacked, forming a three-dimensional porous network structure. This hierarchical structure not only provides abundant exposed active sites but also facilitates mass transfer of the electrolyte and rapid electron transport, which is of significant importance for electrochemical sensing.

**Figure 2:**
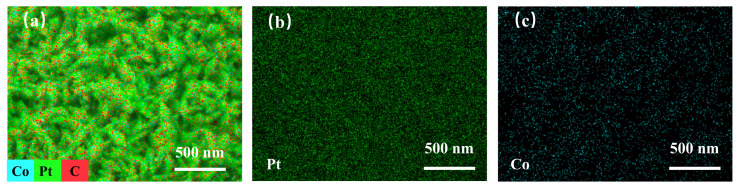
EDS elemental mapping: (**a**) PtCo/CC, (**b**) Pt, (**c**) Co.

Energy dispersive X-ray spectroscopy (EDS) was employed to analyse the elemental composition and distribution of the PtCo/CC electrode. As shown in Figure S1, characteristic peaks corresponding to Pt and Co were clearly observed in the EDS spectrum, confirming the coexistence of both elements in the as-prepared PtCo/CC electrode. The elemental mapping results, presented in Figure 2, reveal that Pt and Co are uniformly distributed across the surface of the carbon fibres, with no obvious elemental segregation observed, preliminarily demonstrating the successful formation of the PtCo alloy.

**Figure 3:**
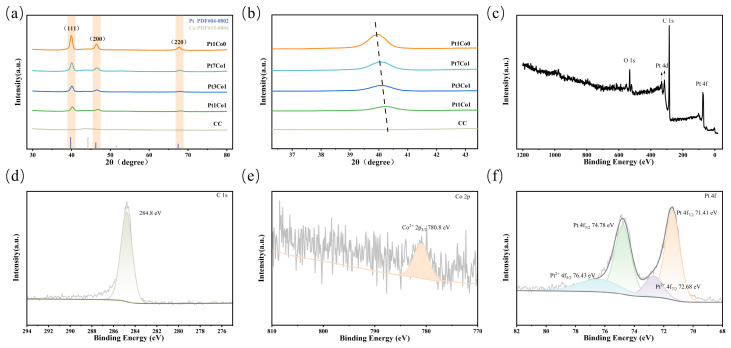
Characterisation of the phase structure and surface chemical states of the PtCo/CC electrode: (**a**) XRD patterns of Pt1Co0/CC, Pt7Co1/CC, Pt3Co1/CC, Pt1Co1/CC and bare CC; (**b**) Enlarged view of the diffraction peak from the Pt(111) crystal plane; (**c**) XPS survey spectrum of the PtCo/CC electrode; the high-resolution XPS spectra of (**d**) C 1s, (**e**) Co 2p, and (**f**) Pt 4f.

To further confirm the formation of the PtCo alloy, the XRD patterns of samples with different Pt/Co molar ratios (Pt1Co0, Pt7Co1, Pt3Co1, and Pt1Co1) were systematically investigated, as shown in Figure 3a. All samples exhibit characteristic diffraction peaks corresponding to the face-centred cubic (FCC) structure of Pt, which are attributed to the (111), (200), and (220) crystal planes of Pt (PDF#04-0802), and no diffraction peaks corresponding to metallic Co (PDF#15-0806) or cobalt oxides are detected [24]. It is worth noting that as the Co content increases, the diffraction peaks gradually shift to higher 2θ angles. As shown in the magnified view of the Pt(111) diffraction peak in Figure 3b, the diffraction peak gradually shifts from 39.9° for Pt1Co0 to 40.1° for Pt7Co1, 40.2° for Pt3Co1, and 40.3° for Pt1Co1. According to Bragg’s law, 2d sinθ = nλ (where d is the interplanar spacing, θ is the diffraction angle, λ is the X-ray wavelength, and n is the diffraction order), an increase in the diffraction angle θ corresponds to a decrease in the interplanar spacing d. This continuous shift to higher angles indicates that the Pt lattice undergoes continuous contraction, which is due to the fact that the atomic radius of Co is smaller than that of Pt, and Co atoms enter the Pt lattice in the form of substitutional doping, resulting in lattice contraction [25]. This phenomenon basically conforms to Vegard’s law, indicating that a substitutional solid solution alloy is formed in the bulk phase.

To further investigate the surface chemical state and electronic structure of the PtCo alloy, XPS was employed to characterise the PtCo/CC electrode. All binding energies were charge-corrected using the C 1s main peak (284.8 eV) as the reference peak. Figure 3c shows the XPS survey spectrum of the PtCo/CC electrode, whilst Figure 3d–f and Figure S2 present the high-resolution XPS spectra of C 1s, Co 2p, Pt 4f, and O 1s. The characteristic peaks of C 1s, O 1s, Pt 4d, and Pt 4f can be clearly observed in Figure 3c. Although the Co 2p signal is weak in the survey spectrum and no distinct characteristic peak is observed, the high-resolution scan (Figure 3e) reveals a peak at 780.8 eV, attributed to Co^2+^ 2p_3/2_, indicating that the surface Co primarily exists in the Co^2+^ oxidation state. As the surface layer of the PtCo alloy is covered by Pt, the Co 2p_1/2_ characteristic peak signal is weak and not clearly evident. Combined with the XRD results, it can be inferred that in the bulk phase, Co atoms have successfully entered the Pt lattice to form a PtCo alloy, while the surface exposed to air has formed a very thin Co^2+^ oxide layer. Figure 3f shows the high-resolution Pt 4f spectrum, which, after peak fitting, can be resolved into two sets of doublets. The doublets with binding energies at 71.41 eV and 74.78 eV are attributed to the metallic Pt 4f_7/2_ and Pt 4f_5/2_, which constitute the major components of the Pt 4f spectrum; the shoulders at 72.68 eV and 76.43 eV can be attributed to the Pt^2+^ 4f_7/2_ and Pt^2+^ 4f_5/2_ oxidation states, likely arising from slight oxidation on the sample surface. The PtCo/CC alloy electrode exists primarily in the metallic Pt state; the high proportion of the metallic state is conducive to enhancing the catalytic activity of the electrode, thereby increasing its electrochemical sensitivity to ammonia nitrogen [26]. It is worth noting that, compared with the standard binding energy of pure metallic Pt (typically around 71.1–71.2 eV), the binding energy of metallic Pt 4f_7/2_ in the PtCo/CC electrode (71.41 eV) exhibits a clear positive shift, indicating that Co doping induces a downshift of the d-band center and thus modulates the electronic structure of Pt [27].

### 3.2. Analysis of the Electrochemical Properties

**Figure 4:**
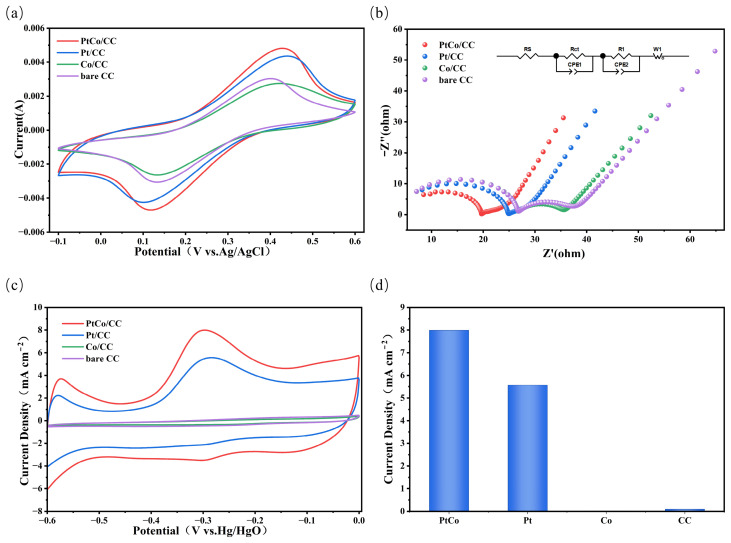
(**a**) CV curves and (**b**) EIS Nyquist plots of different electrodes in 0.1 M KCl using 5 mM [Fe(CN)_6_]^3−/4−^ as the probe; (**c**) CV curves for the ammonia oxidation reaction in 1 M KOH containing 1 mM NH_4_Cl, and (**d**) the corresponding bar chart of peak currents.

To evaluate the electrochemical performance of the different electrodes, cyclic voltammetry (CV) and electrochemical impedance spectroscopy (EIS) tests were first conducted in a 0.1 M KCl solution using 5 mM [Fe(CN)_6_]^3−/4−^ as the probe. As shown in Figure 4a, the PtCo/CC, Pt/CC, Co/CC, and CC electrodes all exhibited reversible redox peaks. Among these, the PtCo/CC electrode exhibited the highest redox peak current, followed by the Pt/CC electrode; the CC electrode showed a weaker peak, while the Co/CC electrode exhibited the weakest. This phenomenon indicates that PtCo/CC possesses a larger active specific surface area and higher conductivity compared to Pt/CC. The weakest peak current of the Co/CC electrode may be attributed to the inherently low electrocatalytic activity of Co under these test conditions, and the fact that its introduction hinders electron transport on the CC substrate surface.

Figure 4b shows the Nyquist plots of the EIS for the four electrodes measured in 0.1 M KCl containing 5 mM [Fe(CN)_6_]^3−^/^4−^. The measurements were performed at the open-circuit potential (OCP) over a frequency range from 0.1 Hz to 1 MHz with an AC perturbation amplitude of 5 mV. The insets depict the equivalent circuit model used for fitting. All impedance spectra exhibit two characteristic time constants, which appear in the Nyquist plots as a main semicircle in the high-frequency region and an additional relaxation process in the mid-frequency region. Accordingly, an equivalent circuit consisting of two parallel R-CPE units in series with a Warburg element, Rs-(Rct//CPE1)-(R1//CPE2)-W, was employed to fit the experimental data. Here, W represents the Warburg impedance, which is the impedance caused by diffusion processes in the electrochemical system. Rs is the solution resistance, Rct corresponds to the charge-transfer process at the electrode/electrolyte interface, and its magnitude is reflected by the diameter of the high-frequency semicircle. The second parallel branch (R1//CPE2) describes the additional relaxation behavior observed in the mid-frequency region. For the ferri/ferrocyanide redox probe system, the mid-frequency time constant is typically associated with surface heterogeneity, interfacial adsorption/desorption processes, and the interface response between the catalytic layer and the substrate [28]. Although the system exhibits two time constants, the high-frequency semicircle remains dominated by the charge-transfer process; therefore, Rct can be used as a key parameter to evaluate the kinetics of interfacial electron transport. The Rct values obtained from equivalent circuit fitting are as follows: PtCo/CC (15.2 Ω), Pt/CC (20.75 Ω), Co/CC (22.05 Ω), and CC (21.95 Ω). The PtCo/CC electrode exhibits the lowest Rct, indicating that the introduction of Co to form a PtCo alloy effectively reduces the charge-transfer resistance, thereby enhancing electron transport efficiency and the electrocatalytic activity at the electrode surface.

**Figure 5:**
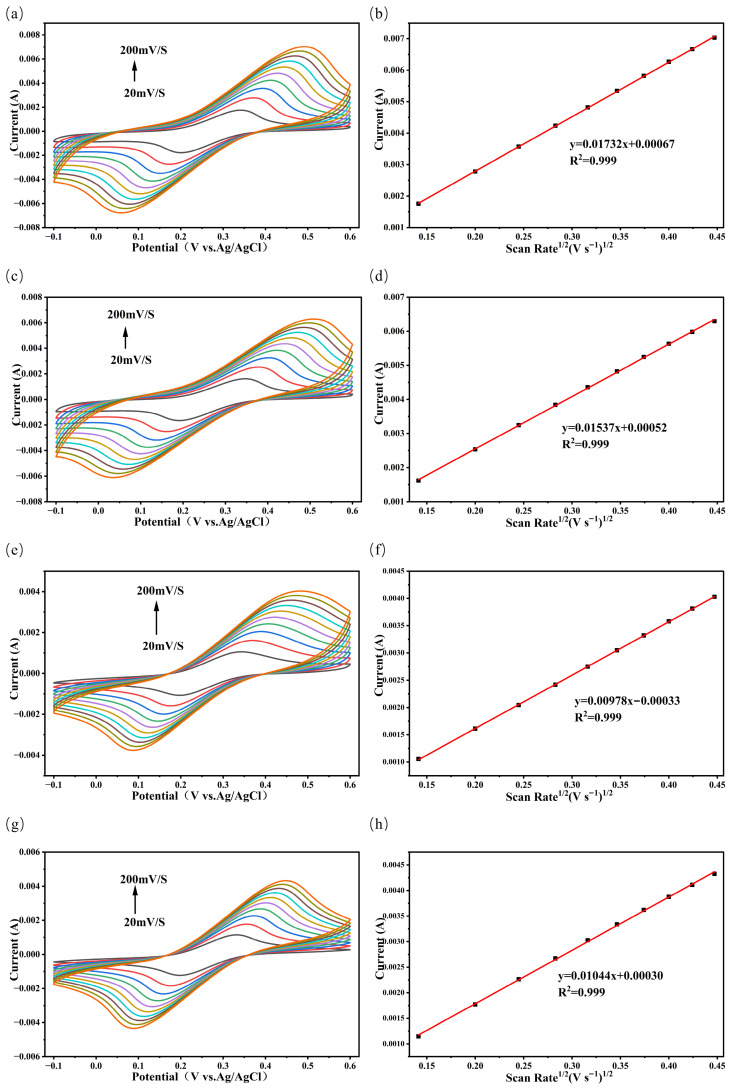
CV curves of the four electrodes at different scan rates (20–200 mV·s^−1^) and the linear fitting relationships between the oxidation peak current and the square root of the scan rate: (**a**,**b**) PtCo/CC, (**c**,**d**) Pt/CC, (**e**,**f**) Co/CC, (**g**,**h**) CC electrodes.

To further determine the electrochemical active specific surface area (ECSA) of the different electrodes, cyclic voltammetry (CV) curves of the aforementioned four electrodes were measured at various scan rates (20–200 mV·s^−1^) in a 0.1 M KCl solution using 5 mM [Fe(CN)_6_]^3−/4−^ as the probe. The linear relationship between the oxidation peak current and the square root of the scan rate was analysed. As shown in Figure 5, the CV curves of the four electrodes—PtCo/CC, Pt/CC, Co/CC, and CC—at different scan rates all exhibit a pair of reversible redox peaks. As the scan rate increased, both the oxidation peak current and the reduction peak current for each electrode gradually increased, and the peak potentials shifted slightly, indicating that the electrode processes were diffusion-controlled. Fitting the relationship between the oxidation peak current (I_pa_) and the square root of the scan rate (v^1/2^), all four electrodes exhibited a good linear relationship. According to the Randles–Sevcik equation [29],

I_pa_ = (2.69 × 10^5^)n^3/2^ACD^1/2^V^1/2^ (1)

where I_pa_ is the oxidation peak current (A), n is the number of transferred electrons (n = 1), A is the electrochemical active specific surface area of the tested electrode (cm^2^), C is the probe molecule concentration (5 × 10^−6^ mol cm^−3^), D is the diffusion coefficient (7.6 × 10^−6^ cm^2^ s^−1^), and V is the scan rate (V s^−1^). Calculations yielded the following ECSA values for each electrode: 4.11 cm^2^ for the PtCo/CC electrode, 3.72 cm^2^ for the Pt/CC electrode, 2.58 cm^2^ for the CC electrode, and 2.35 cm^2^ for the Co/CC electrode. Among these, the PtCo/CC electrode exhibited the largest electrochemical active surface area. This is consistent with the results from the aforementioned CV curves, where it displayed the highest peak current, and from the impedance plots, where it exhibited the lowest charge transfer resistance. This indicates that the formation of the PtCo alloy effectively increases the active surface area of the electrode, facilitating charge transport and mass diffusion during the catalytic reaction.

Based on the excellent electrochemical performance described above, the electrochemical detection capabilities of different electrodes for ammonia nitrogen were further evaluated to investigate the potential application of the PtCo/CC electrode in drinking water quality assessment. The cyclic voltammetry responses and oxidation peak currents of each electrode were tested in a 1 M KOH solution containing 1 mM NH_4_Cl. As shown in Figure 4c, within the potential window of −0.6 V to 0 V (vs. Hg/HgO), both the PtCo/CC and Pt/CC electrodes exhibited distinct ammonia oxidation peak current responses at around −0.3 V, with the PtCo/CC electrode displaying the highest oxidation peak current. In contrast, no distinct ammonia oxidation current response was observed for the Co/CC and CC electrodes within this potential range, indicating that neither possesses effective catalytic activity towards ammonia nitrogen. Figure 4d shows the bar chart of the peak currents for ammonia oxidation for the four electrodes in a 1 mM NH_4_Cl solution. The peak current for the PtCo/CC electrode was 7.988 mA cm^−2^, that for the Pt/CC electrode was 5.566 mA cm^−2^, while the Co/CC electrode exhibited a peak current of 0.0222 mA cm^−2^, and the CC electrode exhibited a peak current of 0.0957 mA cm^−2^. From Figure 4d and the peak current values of the four electrodes, it is clear that the peak current of the PtCo/CC electrode is significantly higher than that of the other three electrodes, indicating that the introduction of Co to form the PtCo/CC alloy significantly enhances the electrocatalytic activity for ammonia oxidation.

According to the Oswin–Salomon and Gerischer–Mauerer mechanisms, the ammonia oxidation reaction primarily follows a stepwise dehydrogenation pathway, in which ammonia molecules adsorb onto Pt active sites and then undergo stepwise dehydrogenation to form various reactive intermediates (*N_2_H_x+y_ or *NH_2_, *NH, and*N), ultimately desorbing as N_2_. The overall reaction equation is

2NH_3(aq)_→N_2,g_ + 6H^+^ + 6 e^−^ (2)

However, pure Pt is susceptible to strong adsorption of intermediates, leading to active site poisoning and limiting its catalytic efficiency. The introduction of Co contracts the Pt lattice and causes a positive shift in the Pt 4f binding energy, inducing a downshift of the Pt d-band centre, thereby weakening the adsorption strength of reaction intermediates on the Pt surface. DFT calculations confirm that this moderate weakening of adsorption can effectively reduce the dehydrogenation energy barriers: Fang et al. studied the ammonia oxidation reaction on PtCo alloys and demonstrated that an appropriate amount of Co significantly lowers the energy barriers for the *NH_2_ → *NH → *N dehydrogenation steps [30]. Consequently, the kinetic hindrance of the reaction is reduced, and the rate of the ammonia oxidation reaction is increased.

Analysis of the electrochemical performance of different electrodes revealed that the PtCo/CC electrode exhibited the highest redox peak current, the lowest charge transfer resistance, and the largest ECSA, along with the optimal catalytic oxidation activity in ammonia nitrogen detection. This outstanding performance can be attributed to two synergistic factors. On one hand, its unique three-dimensional porous nanosheet structure provides a large ECSA, facilitating electrolyte diffusion and electron transport, thereby effectively reducing the charge transfer resistance. On the other hand, the formation of the PtCo alloy induces lattice contraction and downshifts the d-band centre of Pt, which weakens the adsorption strength of reaction intermediates, lowers the kinetic barrier, and further enhances the electrocatalytic ammonia oxidation activity. Together, these structural and electronic effects endow the PtCo/CC electrode with excellent electrochemical performance, demonstrating its strong potential for highly sensitive ammonia nitrogen detection in drinking water quality assessment.

### 3.3. Sensitivity Characteristics of PtCo Electrodes

It has been demonstrated previously that the PtCo/CC electrode exhibits superior electrochemical performance and catalytic activity for ammonia nitrogen. To enhance its sensitivity in the detection of ammonia nitrogen in drinking water, the electrochemical preparation of the PtCo electrode was optimised by varying the alloy ratio, the number of deposition cycles, and the precursor solution concentration.

**Figure 6:**
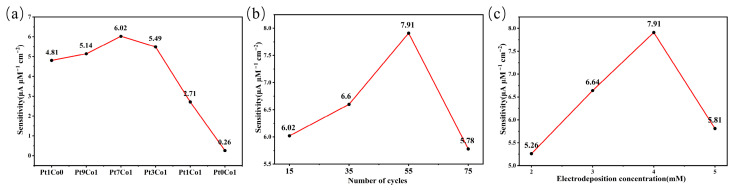
Line graphs of the sensitivity of PtCo/CC electrodes prepared under different deposition conditions towards NH_4_Cl: (**a**) different PtCo ratios; (**b**) different numbers of deposition cycles; (**c**) different precursor concentrations.

In a 1 M KOH electrolyte, Pt1Co0, Pt9Co1, Pt7Co1, Pt3Co1, Pt1Co1, and Pt0Co1 electrodes were used to perform CV tests on 1, 5, 10, 50, and 100 μM NH_4_Cl solutions. The sensitivity of each electrode was calculated by linear fitting of the oxidation peak current versus concentration (Figure S3). As shown in the sensitivity line graph in Figure 6a, the Pt7Co1 electrode exhibited the highest sensitivity (6.02 μA μM^−1^ cm^−2^), significantly higher than that of the other ratios. After determining the optimal alloy ratio, the number of deposition cycles was further optimised. Pt7Co1 electrodes were prepared by depositing 15, 35, 55, and 75 cycles via cyclic voltammetry, and their sensitivity was evaluated under the same concentration gradient. As shown in Figure 6b, the electrode sensitivity was highest (7.91 μA μM^−1^ cm^−2^) when the number of deposition cycles was 55. The corresponding CV curves and linear fitting plots of the oxidation peak current versus concentration are shown in Figure S4. Furthermore, the effect of precursor solution concentration (2, 3, 4, and 5 mM) on electrode sensitivity was investigated. As shown in Figure 6c, the Pt7Co1 electrode prepared with a precursor solution concentration of 4 mM exhibited the highest sensitivity (7.91 μA μM^−1^ cm^−2^); the corresponding CV curves and linear fitting plots of the oxidation peak current versus concentration are shown in Figure S5. The above results demonstrate that the PtCo/CC electrode exhibits the best sensitivity for ammonia nitrogen detection under the preparation conditions of a Pt7Co1 alloy, 55 deposition cycles, and a precursor solution concentration of 4 mM. The optimised PtCo/CC electrode was used for further electrochemical ammonia nitrogen detection.

**Figure 7:**
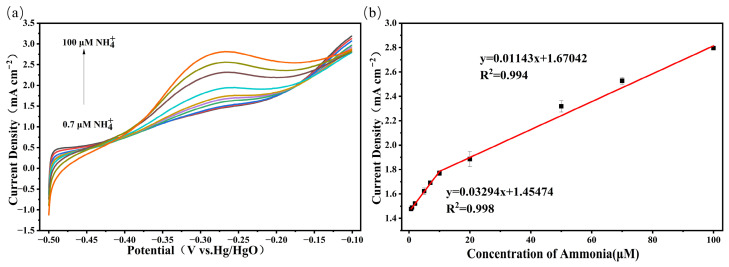
(**a**) LSV curves of the PtCo/CC electrode in 1 M KOH solution containing 0.7–100 μM NH_4_Cl; (**b**) linear fitting of oxidation peak current versus concentration.

The optimal sensitivity of the PtCo/CC electrode for ammonia nitrogen detection obtained by CV testing was 7.91 μA μM^−1^ cm^−2^. To further improve the detection sensitivity, we employed LSV, which features a narrower potential window and a single-scan mode, for subsequent studies on the sensitivity characteristics of ammonia nitrogen detection, aiming to achieve a higher sensitivity. As shown in Figure 7, within the potential window of −0.5 V to −0.1 V (vs. Hg/HgO), the peak current response of the PtCo/CC electrode for ammonia oxidation exhibited a gradually increasing trend at ammonia concentrations ranging from 0.7 to 100 μM, with the peak potential occurring at approximately −0.27 V. Figure 7b shows the linear fit curve of the oxidation peak current versus ammonia nitrogen concentration, exhibiting a good linear relationship in two segments within the 0.7–100 μM concentration range. For ammonia nitrogen concentrations of 0.7–10 μM, the linear fitting equation is Ipa (mA cm^−2^) = 0.03294 C (μM) + 1.45474 (R^2^ = 0.998), with a sensitivity of 32.94 μA μM^−1^ cm^−2^. In the 10–100 μM ammonia nitrogen concentration range, the linear fitting equation is Ipa (mA cm^−2^) = 0.01143 C (μM) + 1.67042 (R^2^ = 0.994), with a sensitivity of 11.43 μA μM^−1^ cm^−2^. The limit of detection (LOD) is one of the key indicators for evaluating an electrode; the calculation formula is LOD = 3σ/s, where σ represents the noise of the blank signal, the standard deviation of the baseline current fluctuation measured at the peak potential in the blank electrolyte over multiple scans, and s is the sensitivity of the PtCo/CC electrode (32.94 μA μM^−1^ cm^−2^). Calculations show that the LOD of the PtCo/CC electrode is 77.9 nM. The PtCo/CC electrode demonstrates excellent detection sensitivity and a low detection limit for ammonia nitrogen, which is significantly lower than the limit specified in China’s “Sanitary Standards for Drinking Water” (GB 5749-2022) (0.5 mg/L, approximately 35.7 μM), thereby fully meeting the detection sensitivity requirements for drinking water quality assessment.

As shown in Table 1, the performance of the PtCo/CC electrode was compared with that of recently reported sensitive electrodes for ammonia nitrogen detection. The PtCo/CC electrode prepared in this study outperformed most previously reported electrode materials in terms of the highest sensitivity (32.94 μA μM^−1^ cm^−2^) and detection limit (77.9 nM), demonstrating excellent detection performance. Although its linear response consists of two ranges (0.7–10 μM and 10–100 μM) and is relatively narrow, this range fully covers the ammonia nitrogen limit specified in China’s “Sanitary Standards for Drinking Water” and is sufficient to meet the practical detection requirements for drinking water quality assessment.

### 3.4. Analysis of the Anti-Interference, Repeatability, Reproducibility and Stability of PtCo/CC Electrodes

To assess the practical application potential of the PtCo/CC electrode for detection in drinking water environments, its anti-interference, repeatability, reproducibility, and long-term stability were systematically investigated.

**Figure 8:**
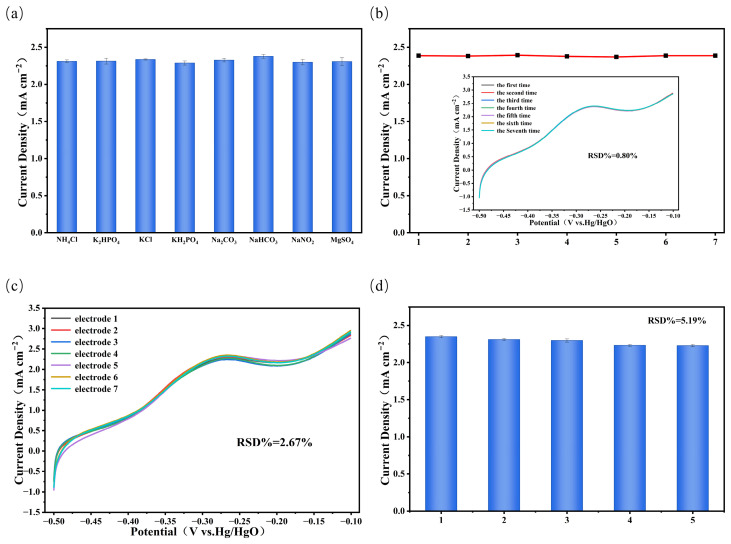
PtCo/CC electrode: (**a**) anti-interference test, (**b**) repeatability test, (**c**) reproducibility test, and (**d**) stability test.

For anti-interference, drinking water has a complex composition and often contains various coexisting ions that may interfere with accurate ammonia nitrogen detection. This study examined the effects of common interferents, including K_2_HPO_4_, KCl, KH_2_PO_4_, Na_2_CO_3_, NaHCO_3_, NaNO_2_, and MgSO_4_ on ammonia nitrogen detection. The experimental procedure was as follows: first, the current response of the ammonia oxidation peak was measured using the PtCo/CC electrode in a 1 M KOH solution containing 50 μM NH_4_Cl; then, after adding the interfering ions at a concentration of 100 μM, the current response of the ammonia oxidation peak was measured again. The experimental results are shown in Figure 8a. The current responses of the ammonia oxidation peak with and without interferents were similar, indicating that the aforementioned common interferents have virtually no effect on the detection. This excellent interference resistance provides an important guarantee for the detection of ammonia nitrogen in actual drinking water samples using this electrode. Regarding repeatability, the PtCo/CC electrode was tested seven consecutive times in a 1 M KOH solution containing 50 μM NH_4_Cl. The experimental results are shown in Figure 8b. The CV curves from the seven consecutive tests exhibited high overlap (inset in Figure 8b), and the current response of the ammonia oxidation peak was stable, with a relative standard deviation (RSD) of 0.80%, demonstrating good repeatability. For reproducibility, seven independently prepared PtCo/CC electrodes were each tested in a 1 M KOH solution containing 50 μM NH_4_Cl. The experimental results are shown in Figure 8c. The CV curves of the seven electrodes almost completely overlapped, with an RSD of 2.67%. Regarding stability, PtCo/CC electrodes were stored for 1 to 5 weeks, and the current response of the ammonia oxidation peak was measured after each storage period. The experimental results are shown in Figure 8d. The current retention rate for the ammonia oxidation peak after 1–5 weeks of storage was 95.8%. Although a slight decrease was observed, the overall stability was good.The PtCo/CC electrode demonstrated excellent performance in terms of interference resistance, repeatability, reproducibility, and stability, indicating its practical application potential for drinking water quality assessment.

### 3.5. Analysis of Actual Water Samples

To evaluate the practical application of the electrode in real drinking water samples, the standard addition method was employed for the detection of ammonia nitrogen in residential drinking water, with results presented in Table 2.

In the experiment, 4 mL of drinking water was mixed with 36 mL of 1 M KOH solution, resulting in a total volume of 40 mL. NH_4_Cl standard solutions were added at concentrations of 1, 3, and 5 μM, respectively. Linear regression was performed with the spiked concentration as the abscissa and the measured total concentration as the ordinate, yielding the regression equation y = 0.075 + 1.045x. The intercept of 0.075 μM represents the initial concentration in the mixed solution. After correcting for the dilution factor, the initial ammonia nitrogen concentration in the residential drinking water was determined to be 0.75 μM. These results indicate that the electrode exhibits good accuracy and reliability in real water samples, meeting the requirements for ammonia nitrogen detection in drinking water quality assessment and demonstrating promising application potential.

## 4. Conclusions

In this study, PtCo alloy nanosheets with a three-dimensional porous structure were grown in situ on carbon cloth using a simple one-step electrodeposition method. A self-supporting PtCo/CC sensitive electrode was successfully fabricated, enabling sensitive detection of ammonia nitrogen in water. The three-dimensional nanosheet structure provides abundant active sites, facilitating electrolyte diffusion and electron transport. The introduction of Co to form an alloy with Pt effectively modulates the geometric and electronic structure of Pt, significantly enhancing the catalytic activity of the electrode. Therefore, under LSV measurements, the PtCo/CC electrode exhibits good sensing characteristics in both linear ranges of 0.7–10 μM and 10–100 μM, with a high sensitivity of 32.94 μA μM^−1^ cm^−2^ and a low detection limit of 77.9 nM in the range of 0.7–10 μM. Furthermore, the electrode demonstrates excellent resistance to interference, as well as excellent repeatability, reproducibility, and stability. Its accuracy and reliability in residential drinking water were further validated through experiments using actual water samples. Thus, this study provides a simple and efficient electrode preparation strategy for highly sensitive ammonia nitrogen detection aimed at drinking water quality assessment.

## Figures and Tables

**Table 1:** Comparison of PtCo/CC electrodes with other electrodes.

Electrode	Sensitivity (μA μM^−1^ cm^−2^)	Linear Range (μM)	LOD (μM)	Method	Reference
PtNi/CC	10.2 2.3	4–50 50–1000	1.154	DPV	[19]
PtZnCu-CC	23.9 4.44	0.5–60 60–1000	0.00837	DPV	[18]
PtAu-PPy-NF	8.2 1.9 0.5	4–50 50–400 400–1000	1.05	DPV	[31]
Ag/Cu_2_O/TNTs	2.937	0.1–101	0.074	i-t	[32]
CuNPs/CC	0.0062 0.0094	5–1325 1325–9425	1.25	i-t	[33]
(Ni,Cu)_2_(OH)_2_CO_3_@CC	3.9 3.13	1–100 100–400	0.62	i-t	[34]
PtZn-CC	21.5 5.89 2.28	1–100 100–400 400–1000	0.02781	DPV	[24]
PtCu-CC	9.5 0.778	0.5–40 40–500	0.00860	DPV	[17]
PtCo/CC	32.94 11.43	0.7–10 10–100	0.0779	LSV	This work

**Table 2:** Detection of ammonia nitrogen in drinking water.

Sample	Initial (μM)	Added (μM)	Found (μM)	Recovery (%)	RSD (%, n = 3)
Drinking water	0.075	1	1.11	103	1.91
		3 5	3.23 5.29	105 104	1.43 2.30

## Data Availability

The data presented in this study are available upon reasonable request from the corresponding author. The data are not publicly available due to institutional policies.

## Supplementary Materials

The following supporting information can be downloaded at https://www.mdpi.com/article/10.3390/s26103103/s1, Figure S1: EDS spectrum of the PtCo/CC electrode; Figure S2: High-resolution XPS spectrum of O 1s; Figure S3: CV curves and linear fitting of oxidation peak current versus concentration for PtCo electrodes with different ratios in 1 M KOH containing 1, 5, 10, 50, and 100 μM NH_4_Cl: (a,a_1_) Pt0Co1; (b,b_1_) Pt1Co1; (c,c_1_) Pt3Co1; (d,d_1_) Pt7Co1; (e,e_1_) Pt9Co1; (f,f_1_) Pt1Co0; Figure S4: CV curves and linear fitting of oxidation peak current versus concentration for PtCo electrodes prepared with different numbers of cyclic voltammetry deposition cycles in 1 M KOH containing 1, 5, 10, 50, and 100 μM NH_4_Cl: (a,b) 15 cycles; (c,d) 35 cycles; (e,f) 55 cycles; (g,h) 75 cycles; Figure S5: CV curves and linear fitting of oxidation peak current versus concentration for PtCo electrodes prepared with different precursor concentrations in 1 M KOH containing 1, 5, 10, 50, and 100 μM NH_4_Cl: (a,b) 2 mM; (c,d) 3 mM; (e,f) 4 mM; (g,h) 5 mM.
