# Supplementary material for: Toward Ultrasensitive Electrochemical Detection of Ammonia Nitrogen in Drinking Water: PtCo Alloy Nanosheet on Self-Supported Carbon Cloth
Source: Sensors (Basel). 2026 May 14;26(10):3103. doi: 10.3390/s26103103 (PMC13210888; doi:10.3390/s26103103)
Supplement: Supplementary file 1 [file sensors-26-03103-s001.zip › sensors-4259074-supplementary.pdf]

Supplementary Material

Toward Ultrasensitive Electrochemical Detection of Ammonia Nitrogen in Drinking Water: PtCo Alloy Nanosheet on Self-Supported Carbon Cloth

Ziyi Zhuang [1], Liang Jia [1], Cong Zhao [2], Zhiyun An [1], Jiameng Chen [1], Chun Zhao [1] and Hui Suo [1,*]

[1] State Key Laboratory of Integrated Optoelectronics, College of Electronic Science and Engineering, Jilin University, Changchun 130012, China; zhuangzy24@mails.jlu.edu.cn (Z.Z.); liangjia25@mails.jlu.edu.cn (L.J.); 17390030123@163.com (Z.A.); chenjm9924@mails.jlu.edu.cn (J.C.); zchun@jlu.edu.cn (C.Z.)

[2] Jilin Province Product Quality Supervision and Inspection Institute, No.2699, YiJu Road,
Changchun 130103, China; zhaocong@jlzjy.org

* Correspondence: suohui@jlu.edu.cn

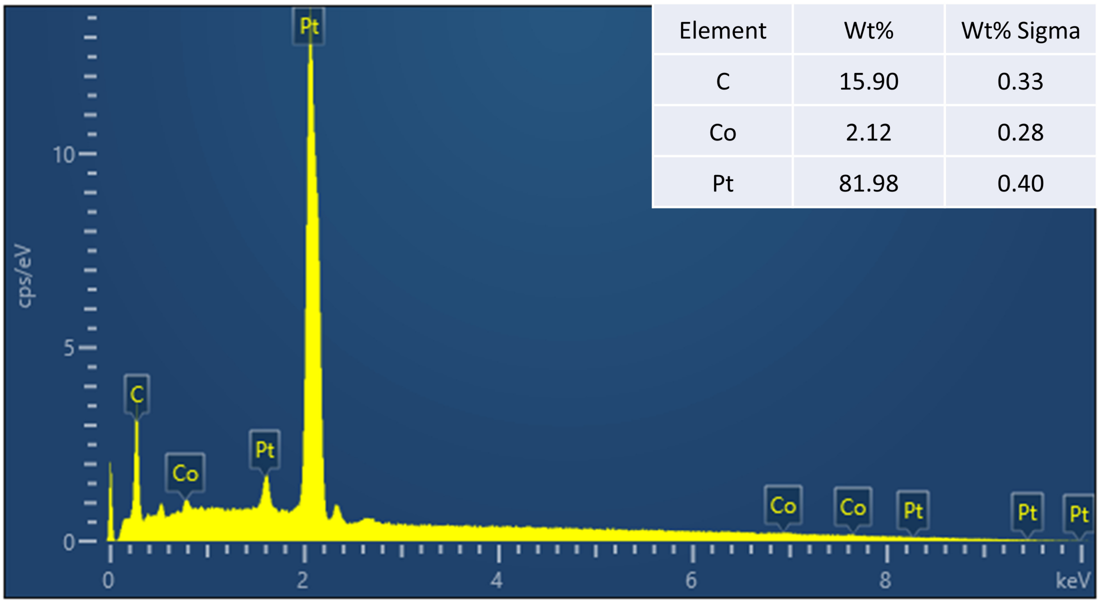

Element	Wt%	Wt% Sigma
C	15.90	0.33
Co	2.12	0.28
Pt	81.98	0.40

Figure S1. EDS spectrum of the PtCo/CC electrode.

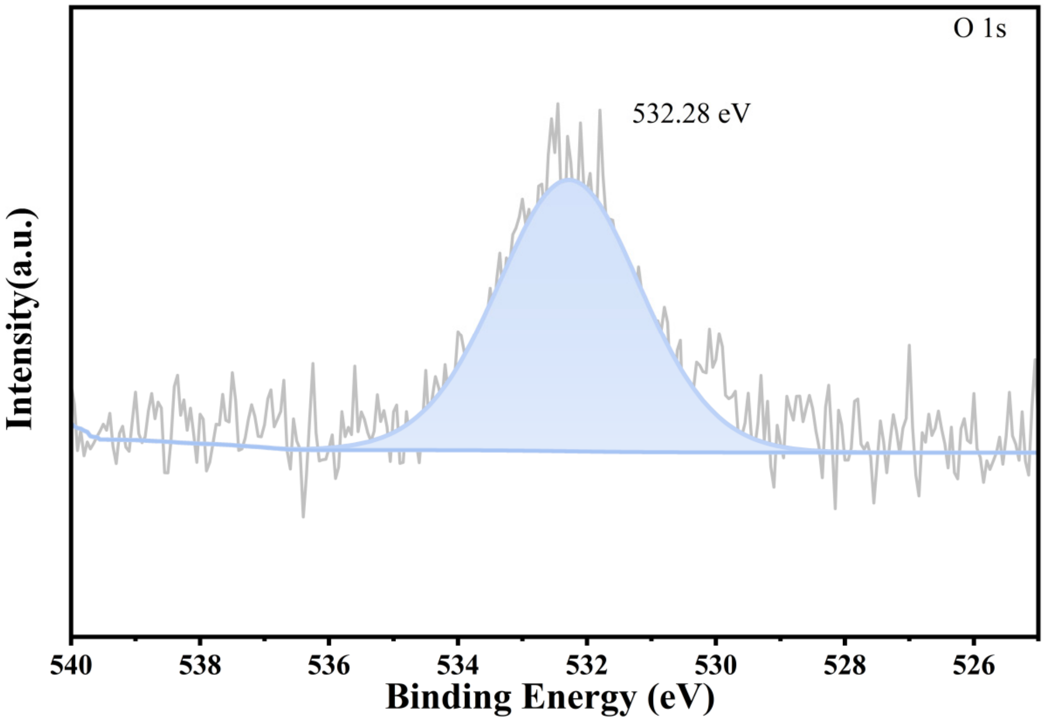

Figure S2. High-resolution XPS spectrum of O 1s.

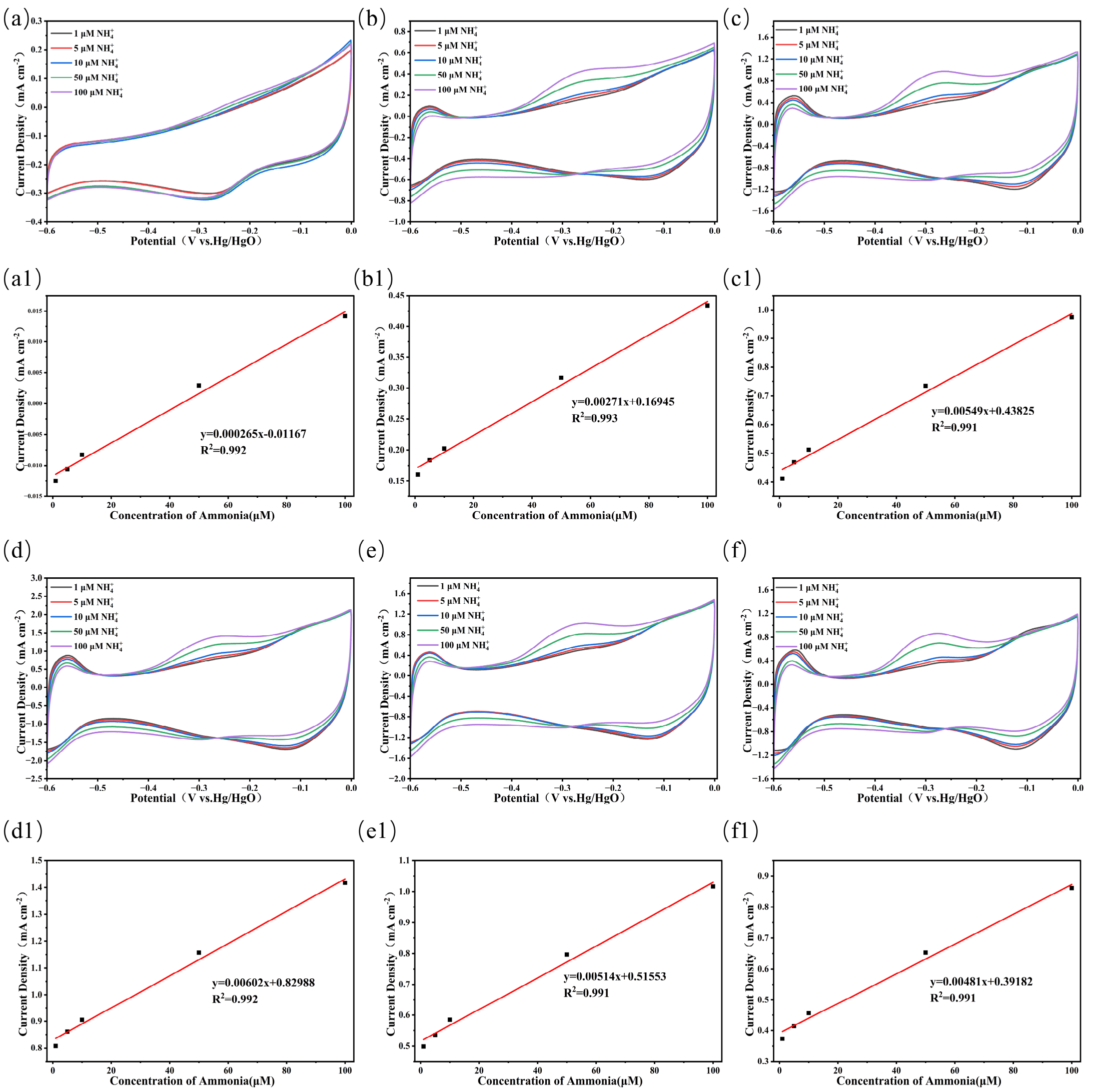

Figure S3. CV curves and linear fitting of oxidation peak current versus concentration for PtCo electrodes with different ratios in 1 M KOH containing 1, 5, 10, 50, and 100 μM NH_4Cl: (a, a_1) Pt0Co1; (b, b_1) Pt1Co1; (c, c_1) Pt3Co1; (d, d_1) Pt7Co1; (e, e_1) Pt9Co1; (f, f_1) Pt1Co0.

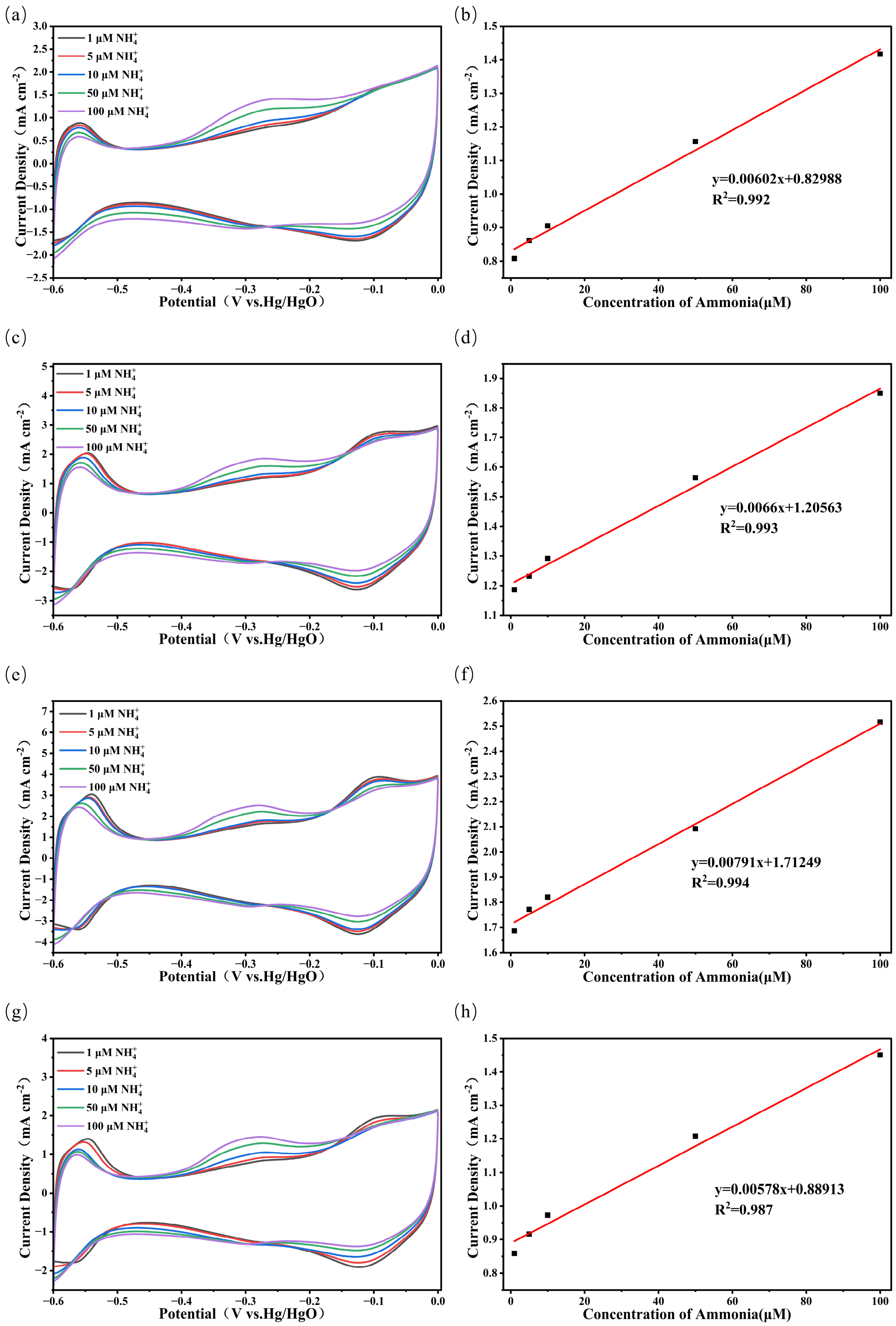

Figure S4. CV curves and linear fitting of oxidation peak current versus concentration for PtCo electrodes prepared with different numbers of cyclic voltammetry deposition cycles in 1 M KOH containing 1, 5, 10, 50, and 100 μM NH_4Cl: (a, b) 15 cycles; (c, d) 35 cycles; (e,

f) 55 cycles; (g, h) 75 cycles.

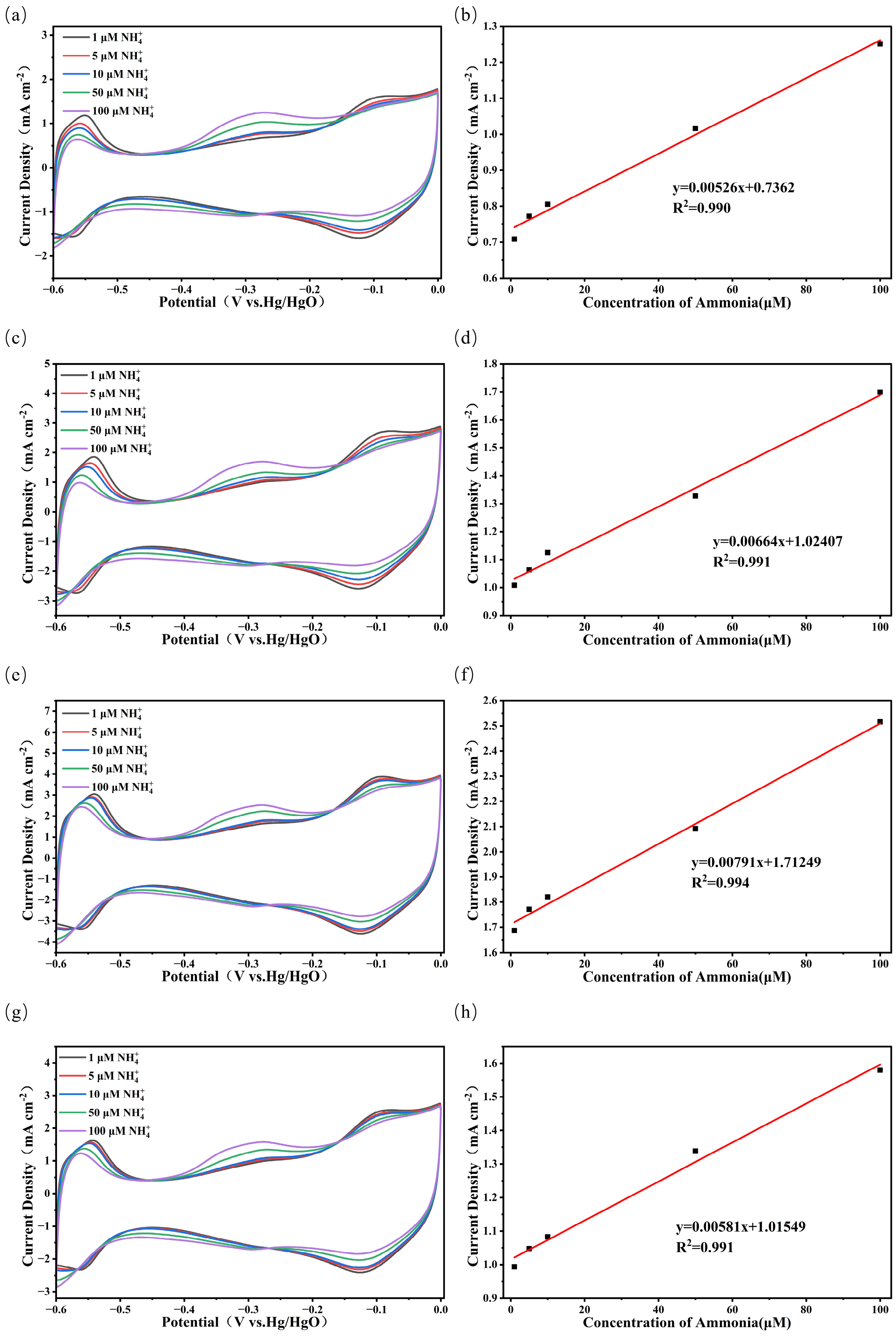

Figure S5. CV curves and linear fitting of oxidation peak current versus concentration for PtCo electrodes prepared with different precursor concentrations in 1 M KOH containing

1, 5, 10, 50, and 100 μM NH_4Cl: (a, b) 2 mM; (c, d) 3 mM; (e, f) 4 mM; (g, h) 5 mM.
